# Peri-implant biomechanical responses to standard, short-wide, and double mini implants replacing missing molar supporting hybrid ceramic or full-metal crowns under axial and off-axial loading: an in vitro study

**DOI:** 10.1186/s40729-017-0094-2

**Published:** 2017-07-13

**Authors:** Lamiaa Said Elfadaly, Lamiaa Sayed Khairallah, Mona Atteya Al Agroudy

**Affiliations:** Fixed Prosthodontics, Cairo University, Giza, Egypt

**Keywords:** Mini implants, Short-wide implants, Standard implants, Axial and off-axial loading, Hybrid ceramics, strain gauge analysis

## Abstract

**Background:**

The aim of this study was to evaluate the biomechanical response of the peri-implant bone to standard, short-wide, and double mini implants replacing missing molar supporting either hybrid ceramic crowns (Lava Ultimate restorative) or full-metal crowns under two different loading conditions (axial and off-axial loading) using strain gauge analysis.

**Methods:**

Three single-molar implant designs, (1) single, 3.8-mm (regular) diameter implant, (2) single, 5.8-mm (wide) diameter implant, and (3) two 2.5-mm diameter (double) implants connected through a single-molar crown, were embedded in epoxy resin by the aid of a surveyor to ensure their parallelism. Each implant supported full-metal crowns made of Ni-Cr alloy and hybrid ceramic with standardized dimensions. Epoxy resin casts were prepared to receive 4 strain gauges around each implant design, on the buccal, lingual, mesial, and distal surfaces. Results were analyzed statistically.

**Results:**

Results showed that implant design has statistically significant effect on peri-implant microstrains, where the standard implant showed the highest mean microstrain values followed by double mini implants, while the short-wide implant showed the lowest mean microstrain values. Concerning the superstructure material, implants supporting Lava Ultimate crowns had statistically significant higher mean microstrain values than those supporting full-metal crowns. Concerning the load direction, off-axial loading caused uneven distribution of load with statistically significant higher microstrain values on the site of off-axial loading (distal surface) than the axial loading.

**Conclusions:**

Implant design, superstructure material, and load direction significantly affect peri-implant microstrains.

## Background

The molars are one of the first teeth to be lost over lifetime; thus, their replacement is frequently needed. Implantation is generally the preferred choice to replace a missing single tooth avoiding vital teeth preparation and bridge fabrication [1].

The mandibular bone loss occurs as knife-edge residual ridge where there is marked narrowing of the labiolingual diameter of the crest of the ridge with a compensatory internal remodeling which sometimes leads to a sharp crest of the ridge which proceeds to low, well-rounded residual ridge [2]. Because of this type of bone loss and the presence of important anatomical areas, the planning of atrophic arches’ posterior sites is normally more complex [3]. The possibilities for patient’s rehabilitation in such limiting situations have involved advanced surgical techniques, such as autogenous bone augmentation and inferior alveolar nerve repositioning. However, these augmentation procedures have some drawbacks such as prolonged time until tooth reconstruction, patient morbidity, and expense. Side effects of bone augmentation include profound edema, pain, and discomfort and possible risks of nerve and blood vessel injury leading to nerve disturbance and hematoma [3, 4].

The use of short implants offer, in relation to the regenerative techniques, several advantages: low cost and treatment length, simplicity, and less risk of complications. An implant is considered to be short if it has a length that is equal to or less than 10 mm [5].

In the last few years, root form implants ranging from 1.8 to slightly more than 2 mm have promoted for long-term use, a task for which the device was approved by the Food and Drug Administration [6].

In situations where there is an inadequate interdental space, reduced interocclusal space, convergent adjacent tooth roots or close proximity of adjacent tooth roots or narrow atrophic osseous contour, mini implants may be appropriate. Nevertheless, when using new available narrow-diameter implants to replace a single molar, two implants could be used even when the distance between the adjacent teeth is smaller [7]. Mini dental implants are minimally invasive since it allows conservative placement of implants in bone without bone grafting and significant trauma and expense for patient and they can be used in patients who would normally be considered high risk (e.g., patients on anticoagulant or steroid therapy). In addition the general dentist can master this technique with minimal training and surgical experience, significantly expanding his armamentarium [6].

There are several factors that affect force magnitudes in peri-implant bone. The application of functional forces induces stresses and strains within the implant prosthesis complex and affect the bone remodeling process around implants [8, 9].

While there are several methods of measuring strain, the most common is with a strain gauge, a device whose electrical resistance varies in proportion to the amount of strain in the device. The most widely used gauge, however, is the bonded metallic strain gauge [10].

Thus, this study aims to evaluate the biomechanical response of the peri-implant bone to standard, short-wide, and two mini implants replacing missing molar with full-metal and Lava ultimate crowns under two different loading conditions using strain gauges.

The hypothesis of this study is that using different implant designs with different superstructure materials would change the peri-implant microstrains.

## Methods

**Fig. 1:**
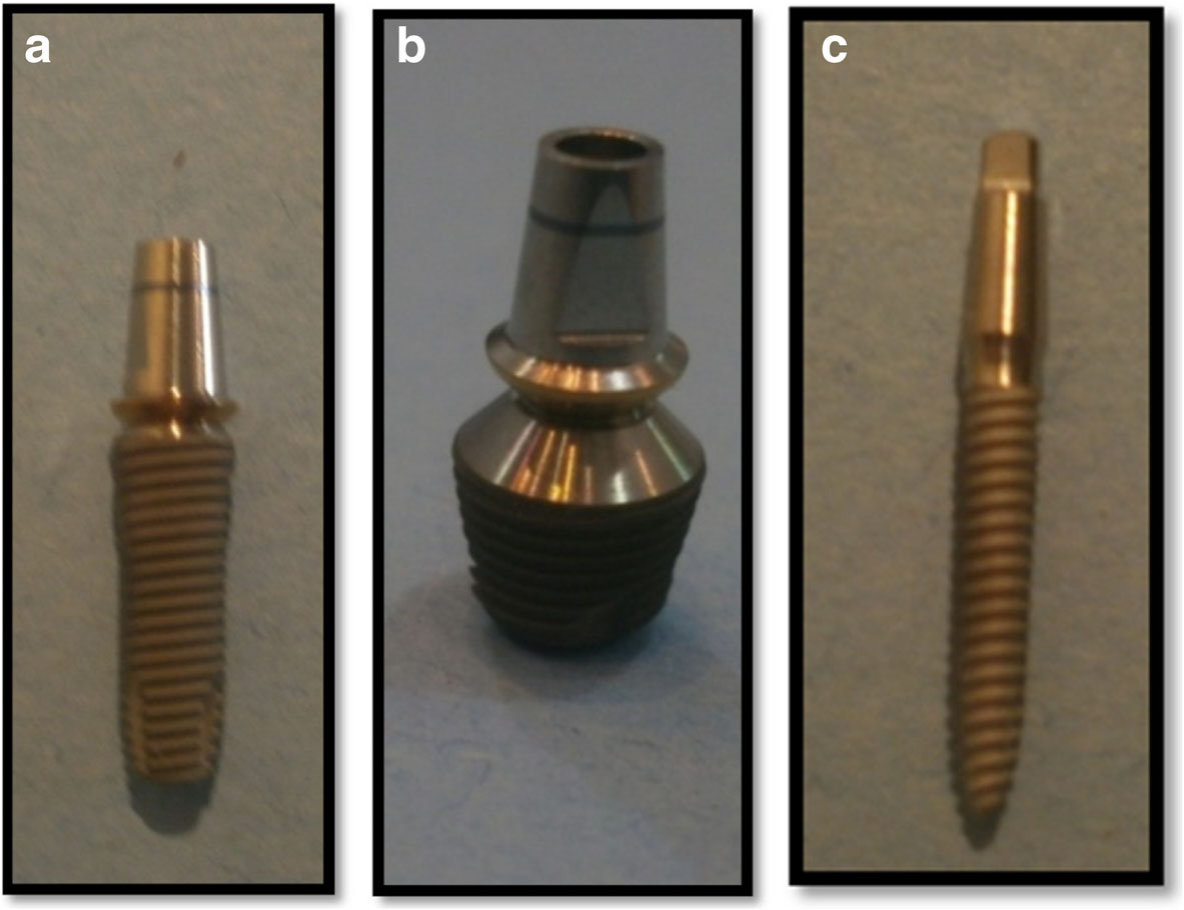
**a** Standard, **b** short-wide, and **c** single-piece mini implants

In the present study, the following materials were used: titanium root form endosseous implants of standard diameter and length (4-mm platform, 3.8-mm diameter,12-mm length, fixture bevel 0.2 mm, Super Line System, Dentium, USA), short-wide implant (7-mm platform, 5.8-mm diameter, 7-mm length, Super Line System, Dentium, Seoul, Korea) with 1.5-mm machined surface and 5.5-mm threaded surface that were fixed and tightened to the internally hexed implants, and 2 one-piece implant with square head mini implants (2.5-mm diameter × 12 mm, Slim Line System, Dentium, Seoul Korea), in addition to titanium implant abutments (straight abutments) with 5.5-mm height and matching width for short-wide and standard implants (5.5 mm and 4.5 mm, respectively) (Fig. 1).

**Fig. 2:**
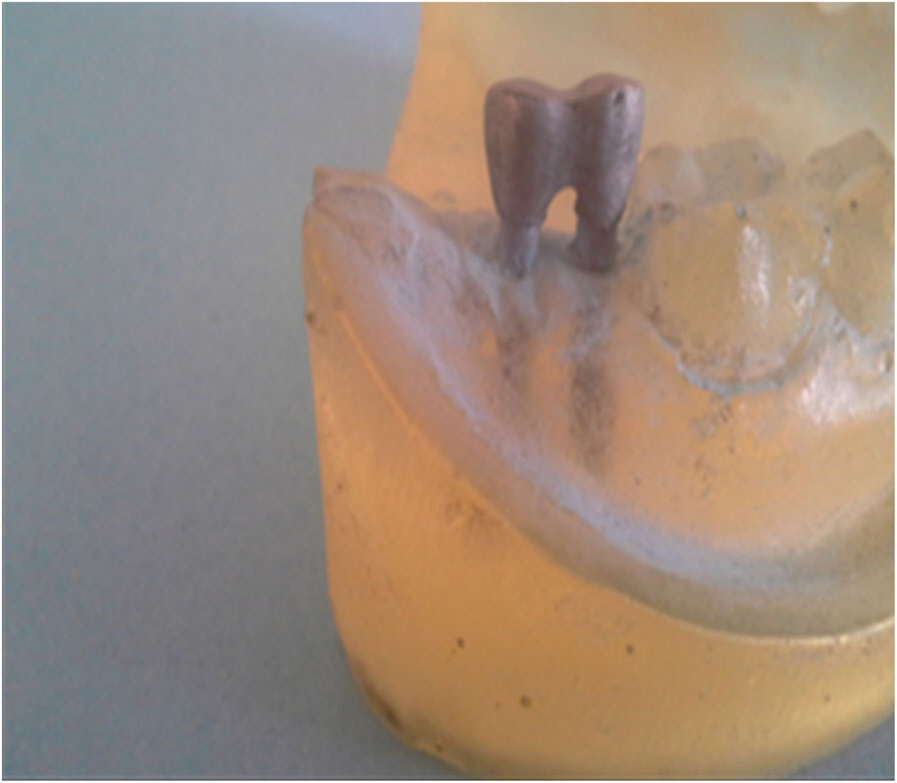
Metal crown supported on two mini implants

**Fig. 3:**
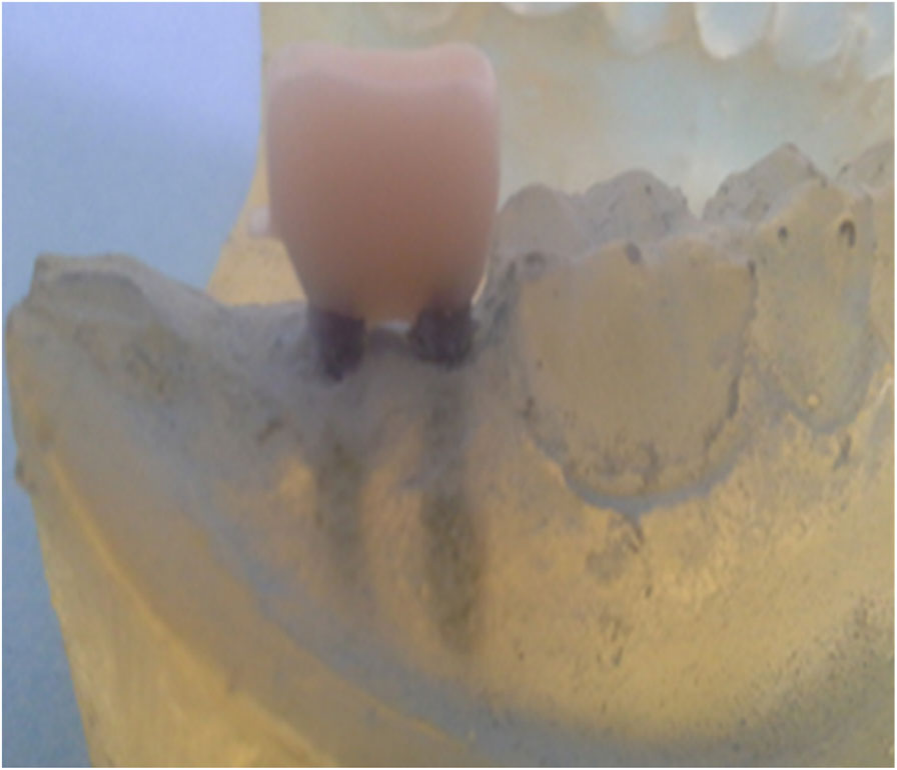
Lava Ultimate Restorative crown on the two mini implants.

Two epoxy resin casts were constructed using epoxy resin material (Transparent Epoxy, Kemapoxy 150, CMB International, Egypt). A dental milling machine (bredent GmbH & Co.KG, Weissenhorner Str. 2, 89250 Senden, Germany) was used to prepare the site for the implant fixtures insertion. The holes were filled with epoxy resin; then, using a dental surveyor (Ramses, Egypt), the implant-abutment units were placed in straight line configuration into the epoxy resin cast which is mounted on surveyor table at zero tilt. The two mini implants were prepared using tapered stone with round end to create a 0.5 chamfer finish line. A total of six crowns were constructed in this study, three full-metal crowns (Kera NH, Deutschland) (Fig. 2), and three hybrid-ceramic (Lava™ Ultimate Restorative, 3M™ ESPE™, Deutschland GmbH) crowns (Fig. 3). They were constructed with standardized dimensions 7-mm height, 7-mm bucco-lingual, and 8-mm mesio-distal width.

A split silicon index was constructed. The first full-metal crown was seated over its corresponding abutment using temporary cement. A duplicating addition silicon impression material was mixed according to the manufacturer’s instructions. The silicon index was split mesiodistally using sharp scalpel into two halves. The other wax patterns were adjusted using this index. The resin nano-ceramic crowns are milled by Computer Aided Design/Computer Aided Manufacturing (CAD/CAM) technology using CEREC inLAB MC XL (Cerec inLab, Sirona dental systems GmbH Fabrikstrasse, Bensheim, Deutschland) with inLab 3D software version 3.88. The restoration was modified to the required dimensions as the metal crowns (7 mm high, 7 mm bucco-lingual, and 8-mm mesio-distal width) by the help of the Cerec grade tool, and the occlusal table was shaped to be non-anatomical.

Each crown was cemented to its corresponding implant-abutment assembly using temporary cement (Cavex Temporary Cement, Cavex, Holland).

**Fig. 4:**
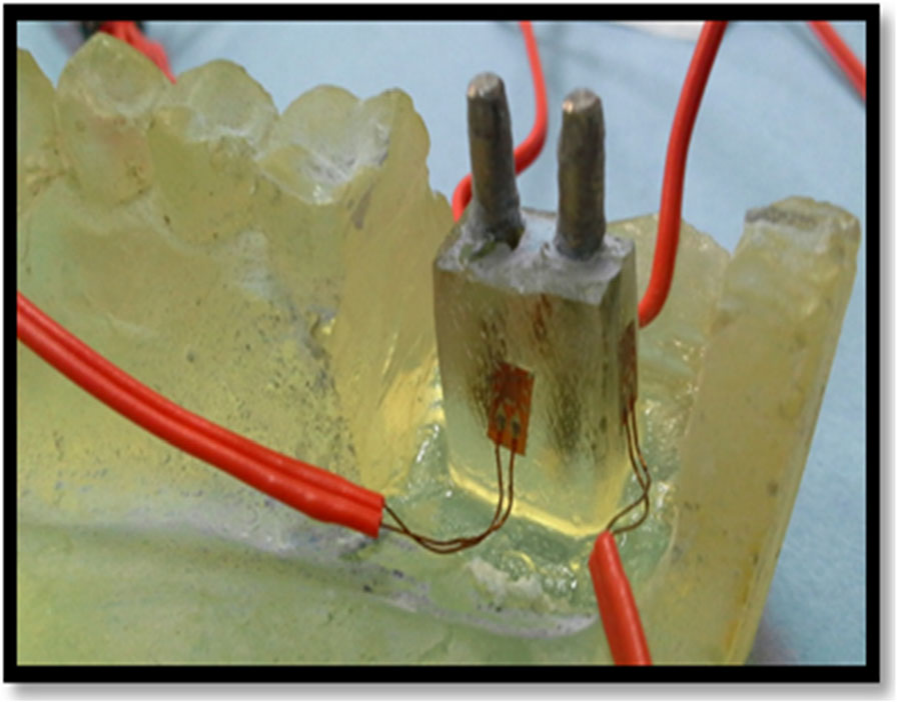
Installation of strain gauges on surfaces of epoxy resin adjacent to mini implants

Each implant received 4 strain gauges (Kowa strain gages, Japan) placed on the mesial, distal, buccal, and lingual surfaces of the epoxy resin adjacent to the implants. At these selected sites, the thickness of the epoxy resin surrounding each implant was reduced to approximately 1 mm and was adjusted to be parallel to the long axis of the implant abutment units using disc and diamond stones (Fig. 4). Electric strain gauges which were 1 mm in length, 2.09 ± 1.0%, and 119.6 ± 0.4 Ω were bonded to their corresponding sites using cyanoacrylate adhesive (Amir, Egypt).They were bonded in a vertical position parallel to the implant bodies and held in place for about 5 min using adhesive tape. The lead wire from each active strain gauge was connected to a multichannel strain meter to register the microstrains transmitted to each strain gauge.

Functional loads of 300 N were applied to the crowns using computerized testing machine (Lloyds LR5K Plus Advance Universal Testing System, Johnson Scale CO., Inc). The machine is computer controlled by the Nexegen ver 4.3 software which permits the collection of data.

**Fig. 5:**
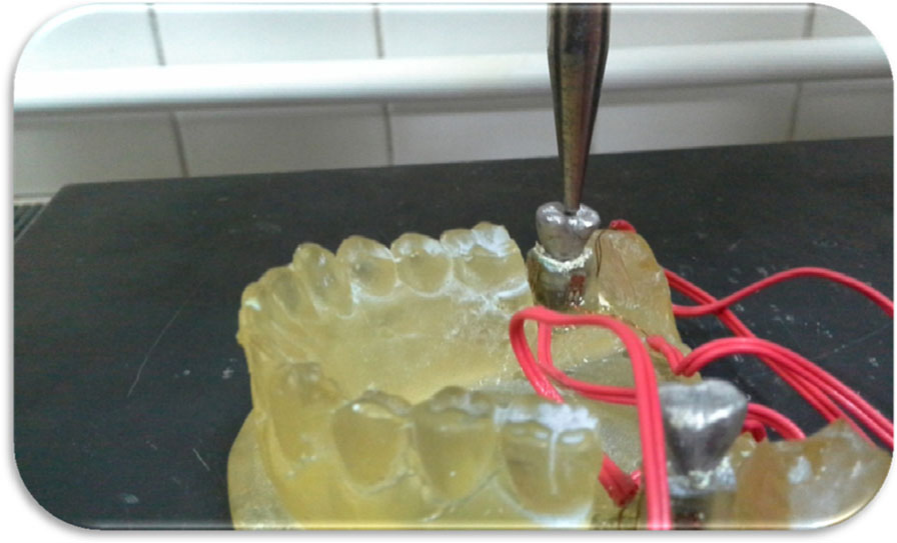
Loading of implant axially

**Fig. 6:**
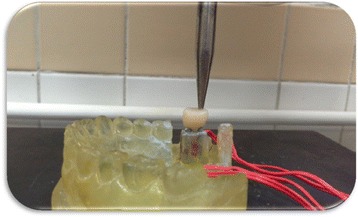
Loading of implant off-axially

Two types of static axial loads were applied with 0.5 mm/min speed. The first load was 300 N applied axially in the position of the centric fossa of each crown Fig. 5 while the second load was 300 N applied 3 mm off-axial distally Fig. 6. The B/L and M/D strains were recorded separately for each strain gauge. Records were repeated five times, allowing the strain indicator to recover to 0 strain before reloading. A fundamental parameter of the strain gauge is its sensitivity to strain, expressed quantitatively as the gauge factor (GF). Gauge factor is defined as the ratio of fractional change in electrical resistance to the fractional change in length (strain) [10]:

$$ \mathrm{G}\mathrm{F}=\frac{\Delta \mathrm{R}/\mathrm{R}}{\Delta \mathrm{L}/\mathrm{L}}=\frac{\Delta \mathrm{R}/\mathrm{R}}{\upvarepsilon} $$

Data were presented as mean and standard deviation (SD) values. Data were explored for normality by checking data distribution and histograms, calculating mean and median values, and finally using Kolmogorov-Smirnov and Shapiro-Wilk tests of normality. Stress data showed non-parametric distribution, so the Kruskal-Wallis test was used to compare between the types of implants. The Mann-Whitney *U* test with Bonferroni’s adjustment was used for pair-wise comparisons when the Kruskal-Wallis test is significant. The Mann-Whitney *U* test was also used to compare between the two crown types. The Wilcoxon signed-rank test was used to compare between axial and off-axial loads.

The significance level was set at *P* ≤ 0.05. Statistical analysis was performed with IBM (IBM Corporation, NY, USA) SPSS (SPSS, Inc., an IBM Company) Statistics Version 20 for Windows.

## Results

### Effect of implant design on peri-implant microstrains

Results revealed that standard implant showed the statistically significantly highest mean microstrain values (3362.4 ± 757.4 μɛ). Double mini implant showed statistically significantly lower mean microstrain values (801.6 ± 251.4 μɛ), while short-wide implant showed the statistically significantly lowest mean microstrain values (697.6 ± 79.7 μɛ), with a *P* value <0.001 (Table 1).

**Table 1:** Descriptive statistics and results of comparison between microstrains induced with different implant design regardless of other variables (collective microstrains)

Standard		Short-wide		Double mini		*P* value
Mean	SD	Mean	SD	Mean	SD	
3362.4^a^	757.4	697.6^c^	79.7	801.6^b^	251.4	<0.001*

Different superscripts in the same row are statistically significantly different

*Significant at *P* ≤ 0.05

### Effect of implant design with different crown material types under different loading directions on overall peri-implant microstrains

#### Under axial loading

The highest statistically significant microstrains were obtained with standard implant supporting Lava Ultimate and metal crowns (3826.5 ± 723.5 μɛ and 2922.5 ± 218.6 μɛ, respectively) while the lowest statistically significant microstrains were obtained with double mini implant supporting metal crown (238.2 ± 32.3 μɛ),with a *P* value <0.001 (Table 2).

**Table 2:** Descriptive statistics and results of comparison between microstrains induced with different implant designs with each crown material (overall microstrains)

Load	Crown type	Standard		Short-wide		Double mini		*P* value
		Mean	SD	Mean	SD	Mean	SD	
Axial	Lava Ultimate	3826.5	723.5	991.5	101.4	939.8	78.3	<0.001*
	Metal	2922.5	218.6	730.8	84.9	238.2	32.3	<0.001*
Off-axial	Lava Ultimate	4286.4	70.9	382.3	41.1	1137.6	86.9	<0.001*
	Metal	2414.4	167.6	685.8	118.4	890.8	118.5	<0.001*

*Significant at *P* ≤ 0.05

#### Under off-axial loading

The highest statistically significant microstrains were obtained with standard implant with Lava Ultimate and metal crowns (4286.4 ± 70.9 μɛ and 2414.4 ± 167.6 μɛ, respectively) while the lowest microstrain was obtained with short-wide implant with Lava Ultimate and metal crowns (382.3 ± 41.1 μɛ and 685.8 ± 118.4 μɛ, respectively), with a *P* value <0.001 (Table 2).

### The effect of crown material type regardless of other variables

Results revealed that implants supporting Lava Ultimate crowns showed statistically significantly higher mean microstrain values (1927.3 ± 1536.6 μɛ) than those supporting metal crowns (1313.7 ± 973.1 μɛ),with a *P* value <0.001 (Table 3).

**Table 3:** Descriptive statistics and results of comparison between microstrains induced by the two crown materials regardless of other variables (collective microstrains)

Lava Ultimate crowns		Metal crowns		*P* value
Mean	SD	Mean	SD	
1927.3	1536.6	1313.7	973.1	<0.001*

*Significant at *P* ≤ 0.05

### Effect of load direction on peri-implant microstrains

Results revealed that there was no statistically significant difference between axial loading (1608.2 ± 1339.0 μɛ) and off-axial loading (1632.9 ± 1356.4 μɛ) of different implant designs supporting different types of crown materials (Tables 4 and 5).

**Table 4:** Descriptive statistics and results of comparison between microstrains induced by the two load directions regardless of other variables (collective microstrains)

Axial		Off-axial		*P* value
Mean	SD	Mean	SD	
1608.2	1339.0	1632.9	1356.4	0.948

*Significant at *P* ≤ 0.05

**Table 5:** Descriptive statistics and results of comparison between microstrains induced by the two load directions with each implant design and crown material (overall microstrains)

Crown	Implant type	Axial		Off-axial		*P-*value
		Mean	SD	Mean	SD	
Lava Ultimate	Standard	3826.5	723.5	4286.4	70.9	<0.001*
	Short	991.5	101.4	382.3	41.1	<0.001*
	Mini	939.8	78.3	1137.6	86.9	<0.001*
Metal	Standard	2922.5	218.6	2414.4	167.6	<0.001*
	Short	730.8	84.9	685.8	118.4	<0.001*
	Mini	238.2	32.3	890.8	118.5	<0.001*

*Significant at *P* ≤ 0.05

### Effect of load direction on different implant designs with different crown materials on overall peri-implant microstrains

#### With Lava Ultimate crowns

In standard as well as double mini implants, off**-**axial loading showed statistically significantly higher mean microstrain values (4286.4 ± 70.9 μɛ and 1137.6 ± 86.9 μɛ, respectively) than axial loading (3826.5 ± 723.5 μɛ and 939.8 ± 78.3 μɛ, respectively). While with short-wide implant, axial loading showed statistically significantly higher mean microstrain values (991.5 ± 101.4 μɛ) than off-axial loading (382.3 ± 41.1 μɛ).

#### With metal crowns

In standard as well as short-wide implants, axial loading showed statistically significantly higher mean microstrain values (2922.5 ± 218.6 μɛ and 730.8 ± 84.9 μɛ, respectively) than off-axial loading (2414.4 ± 167.6 μɛ and 685.8 ± 118.4 μɛ), respectively. While with double mini implants, off-axial loading showed statistically significantly higher mean microstrain value (890.8 ± 118.5 μɛ) than axial loading (238.2 ± 32.3 μɛ).

## Discussion

To replace a missing lower molar in compromised ridge, different treatment options were suggested, using either a standard size implant with surgical procedures, short-wide implant, or two mini implants. Concerning the use of mini implant, splinted multiple implants increase the surface area that interfaces with the bone to lessen the per square millimeters of force borne by the bone [11]. The implant design affects the magnitude of stresses and their impact on the bone implant interface. Screw-shaped implants were used due to the fact that threads of implants decomposes axial load into two components which are parallel and perpendicular to the plane of threads, since it was proven that distribution of the same force over a larger surface leads to lowering of the stresses [12]. The epoxy resin was used to simulate bone matrix as it has mechanical properties similar to those of trabecular bone, Young’s modulus equals 3000 MPa [13].The amount of load used in this experiment, 300 N, was based on the study by Mericske-Stern et al. [14]. The rational for applying the loads on flat occlusal surfaces was to compare axial loading with absolute off-axial loading. As in the presence of cusp inclination, an additional horizontal load would be applied depending on the amount of cusp inclination, thus leading to reduction of the amount of vertical load transferred to the implants [15]. During clinical loading of an implant, the direction of loading is rarely along its central long axis, so the applied occlusal force is frequently in a direction that creates a lever arm, causing off-axial and bending moments in bone [16]. So, by measuring axial and off-axial loads, it was possible to evaluate the load transfer characteristics not only under the regular masticatory forces but also under the extreme load levels, such as those that occur during parafunction [12].

Previous studies have shown that direct correlations exist between microstrain magnitudes and bone stability/instability conditions. This has been summarized by Frost, when bone is loaded below about 2000 microstrains, bone can easily repair what little microdamage occurs. Yet, when pathologic overloading occurs (over 4000 microstrains), stress and strain gradients exceed the physiologic tolerance threshold of bone and cause micro-fractures at the bone-implant interface [17]. Thus, the maximum normal stress criterion, 3000 μɛ, was used to evaluate the extent of the regions where the normal stresses were beyond the allowable tensile and compressive values in the cortical bone [12].

So the implant design that experienced the least overall amount of strain was thought to represent the best design, at least in terms of stress distribution.

Results revealed that all implant designs with different superstructure materials and under different loading conditions resulted in peri-implant microstrain values which were within the physiologic loading zone, below 3000 μɛ, except for the standard sized implants supporting Lava Ultimate crowns under axial and off-axial loading.

Regarding the effect of implant design on peri-implant microstrains induced in the present study, standard diameter implant showed the highest microstrain values regardless of other variables (3362.4 ± 757.4 μɛ). Microstrain value exceeded the physiologic limit in the standard implant supporting Lava Ultimate crowns. That was in approval with Balshi et al. who stated that replacing a lost molar with only one implant represents a biomechanical challenge [18]. This might be attributed to the differences in the size and morphology of natural tooth roots and the standard size implants (3.75 or 4 mm), thus providing insufficient support [19]. In the present study regardless of other variables, double mini implant showed statistically significantly lower mean microstrains (801.6 ± 251.4 μɛ) than standard implant. Moreover, the double mini implant showed statistically significantly lowest microstrain values with metal and Lava Ultimate crowns under axial loading. Under off-axial loading, it also showed statistically significant lower microstrains value than standard implant. Moreover, the use of two implants provides more surface area for osseointegration and spreads the occlusal loading forces over a wider area while reducing the potential bending forces that would exist in a single-implant molar restoration [1, 18, 20, 21]. The one-piece design of small-diameter implants (1.8–3.0-mm diameter) provides strength to the implant in comparison with small diameter two-piece implants [22]. According to Misch [23], a solid implant with a 1.23-mm diameter has the same resistance to bending fracture as the annulus region of a 3.75-mm traditional design. Moreover, a solid 3-mm implant has an approximately 340% increase in moment of inertia over the 3.75-mm traditional two-piece root form at the annulus position. Generally, short-wide implant resulted in the lowest microstrain values in comparison with the other two implant designs. The reduced strains associated with wider implants may be due to the increased structural capacity and the enlarged resin-implant contact area offered by these implants, resulting in lower torque effect in conjunction with off-axial loading [20]. Accordingly, Balshi et al. [18] indicated that a molar crown supported by a standard or narrow size implant can easily introduce large bending moments to bone because the dimensions of crown are usually greater than the diameter of the implants. Thus, the wide implant is suggested for placement at the molar region to reduce the possibility of overload. The area that transfers the compressive and tensile loads to bone, that is, functional surface area, was proved to be confined to the crestal 5–7 mm [24–27]. Thus, short implant with a wider diameter provides both improved primary stability and increased functional surface area as it allows engagement of a maximal amount of bone and better distribution of stress in the surrounding bone compared with increases in implant length [28–32]. An increase in diameter by 1 mm will increase the surface area by 30–200% depending on the implant design [33]. Moreover, according to Misch [34], the large-diameter implants which have a larger prosthetic platform exhibit less force transmission.

Regarding the effect of direction of loading on induced microstrains, it was shown that changing the position of occlusal loading had a considerable effect on the amount of distribution of stresses where axial loading generated even distribution of load around the implant in comparison to off-axial loading where stresses were more pronounced in the area of load application. This might be due to the increase of the horizontal component of the applied load which was stated to generate an increase in the moment and eventually an increase in the compressive load on the side of applied force, to a level higher than the compressive load generated by only the vertical component of the force generated by axial loading [12]. Yet, regardless of other variables, there was no statistically significant difference between axial and off-axial loads. Regarding the off-axial in comparison to axial loading, a particularly high risk of traumatic overload occurred with the standard single-unit implant restoration because the restoration itself is usually wider than the implant, creating the potential for a cantilever effect with high bending moments, in off-axial loading [35]. So the loaded side implants bear more stresses on its distal part due to bending moments of the cantilever on the restorations which in turn transfer more stresses to the peri-implant bone at this side [36]. Flanagan [37] stated that mini implant smaller surface area and volume places more force per square millimeter against the encasing bone than larger diameter implants. So, mini implants may be best used in multiples to resist off-axial forces to prevent metal fatigue and fracture. According to Kheiralla and Younis [12], off-axial loading of single mini implant (3-mm diameter) supporting single molar crown induced mean microstrains value higher than the physiologic limit while in this study, the mean microstrain value of double mini implant supporting metal and Lava Ultimate crowns under off-axial loading were within the physiologic limit (890.8 μɛ and 1137.6 μɛ, respectively).This is in accordance with Balshi et al. [38] who stated that two implants can basically eliminate MD bending and that this situation can enable double implants to induce even less load magnification than a wide diameter implant. In this study, although short-wide implants showed mean peri-implant microstrains under axial loading higher than off-axial loading, axial loading of short-wide implant resulted in compression microstrains in all surfaces in case of metal and lava ultimate crowns, indicating that microstrains were distributed almost equally on all surfaces under both axial and off-axial loading. In this study, it was noticed that short-wide implant showed lowest off-axial loading in comparison with standard and double mini implants. Javris [39] emphasized the biomechanical advantage of wide-diameter implants, particularly in reducing the magnitude of stress delivered to the various parts of the implant. The diameter of the implant is related to the bending fracture resistance or moment of interia, and the increase in diameter decreases the risk of fracture to the power of four, provided all other geometric features remain the same. As a result, wider diameter implants may be used when offset loads (cantilevers) or greater stress conditions (i.e., parafunction, molar regions) exist. Moreover, this feature allows better distribution of occlusal forces [33, 40–42]. Rangert [43] considers that wide, single implants are the best choice to resist lateral forces. An increased width of an implant may decrease offset loads, thus increasing the amount of the implant-bone interface placed under compressive loads [34].

Regarding the effect of superstructure material on induced microstrains, generally, different implant designs supporting Lava Ultimate crowns showed higher mean microstrain values(1927.3 ± 1536.6 μɛ), in comparison with those supporting metal crowns (1313.7 ± 973.1 μɛ).Theoretical considerations [44, 45] and in vitro experiments [46–49] suggest that an occlusal material with a low modulus of elasticity such as acrylic resin might dampen the occlusal impact forces, thereby decreasing its effect on the bone-implant interface. Various methods have been proposed to address the issue of reducing implant loads. Yet, all these studies were in contradiction with the results of our study. In accordance with our study, in vitro studies suggested a better load distribution from high elastic modulus material [44]. It has been suggested that stiffer prosthesis materials might distribute the stress more evenly to the abutments and implants [50]. Duyck et al. [51], in an in vivo study, demonstrated a better distribution of bending moments (in contrast to acrylic) when metal was used as prosthesis material in cantilevered or longer span prostheses. Stegaroiu et al. [52, 53] demonstrated that stresses on the bone-implant interface using resin prostheses were similar to or higher than models using gold or porcelain. Desai et al. [54] concluded by 3D FEA that PFM crown reduced stresses around the implant as compared to acrylic crown. Single crowns in the present study were loaded with vertical loads that increased with time. Most in vitro studies on the influence of superstructure materials on the strain transmitted through the implant have been conducted under impact forces. However, it was proven that the mandible is decelerated prior to tooth contact, in contrast to impact forces. Since it has been suggested that impact forces occur only accidently during mastication, the shock-absorbing effect of resilient materials that has been reported under this loading in vitro might not be relevant during most actual mastication. Consequently, the use of resilient material as a superstructure material, though previously recommended to ensure shock protection of the implant-bone interface, does not seem to ease the strain in the bone around implants under simulated masticatory cycles and static loading [52, 53, 55, 56].

## Conclusions

Within the limitations of this in vitro study, the following conclusions could be drawn:

1. Implant design, superstructure material, and load direction significantly affect peri-implant microstrains.
2. The recorded compressive and tensile microstrains for the tested designs were within the physiologic loading range, as they did not exceed the compressive or tensile strength of the bone-implant interface, which is more than 3000 microstrains except for the standard sized implant supporting Lava Ultimate crowns under both loading directions.
3. Off-axial loading leads to uneven distribution of loads, in standard diameter implant, due to the cantilever effect, which caused microstrain values exceeding the physiologic limit, thus causing clinical failure over time.
4. Use of splinted double mini implants and short-wide implant to restore missing mandibular molar reduces cantilever effect which leads to lowering of peri-implant microstrains under off-axial loading.
5. Usage of full-metal crown implant superstructure reduces the peri-implant microstrain values compared to using Lava Ultimate crowns.

## References

[1] Mazor Z, Lorean A, Mijiritsky E, Levin L (2012). Replacement of a molar with 2 narrow diameter dental implants. Implant Dent.

[2] Atwood D (1963). Postextraction changes in the adult mandible as illustrated by micrographs of midsagittal sections and serial cephalometric roentgenograms. J Prosthet Dent.

[3] Felice P, Pellegrino G, Checchi L, Pistilli R, Esposito M (2010). Vertical augmentation with interpositional blocks of anorganic bovine bone vs. 7-mm-long implants in posterior mandibles: 1-year results of a randomized clinical trial. Clin Oral Implants Res.

[4] Shatkin T, Petrotto C (2012). Mini dental implants: a retrospective analysis of 5640 implants placed over a 12-year period. Compend Contin Educ Dent.

[5] Monje A, Chan HL, Fu JH, Suarez F, Galindo-Moreno P, Wang HL. Are short dental implants (<10 mm) effective? A meta-analysis on prospective clinical trials. J Periodontol. 2013;84(7):895–904. 10.1902/jop.2012.120328 22917114

[6] Christensen G (2006). The ‘mini’-implant has arrived. J Am Dent Assoc.

[7] Flanagan D, Mascolo A (2011). The mini dental implant in fixed and removable prosthetics: a review. J Oral Implantol.

[8] Bidez M, Misch C (1992). Force transfer in implant dentistry: basic concepts and principles. J Oral Implantol.

[9] Branemark P, Zarb G, Albrektsson T (1987). Tissue-integrated prosthesis. Osseointegration in clinical dentistry.

[10] Strain gauge measurement—a tutorial. 1998

[11] Flanagan D (2008). Fixed partial dentures and crowns supported by very small diameter dental implants in compromised sites. Implant Dent.

[12] Kheiralla L, Younis J. Peri-implant biomechanical responses to standard, short-wide and mini implants supporting single crowns under axial and off-axial loading (An In-Vitro study). J Oral Implantol. 2014;40(1):42-52. 10.1563/AAID-JOI-D-11-00102 22208865

[13] Renouard F, Rangert B (1999). Risk factors in implant dentistry.

[14] Mericske-Stern R, Assal P, Merickse E, Ing W (1995). Occlusal force and oral tactile sensibility measured in partially edentulous patients with ITI implants. Int J Oral Maxillofac Implants.

[15] Bozkaya D, Muftu S, Muftu A (2004). Evaluation of load transfer characteristics of five different implants in compact bone at different load levels by finite elements analysis. J Prosthet Dent.

[16] Barbier L, Vander SJ, Krzesinski G, Schepers E, Van der Perre G (1998). Finite element analysis of non-axial versus axial loading of oral implants in the mandible of the dog. J Oral Rehabil.

[17] Saime S, Murat C, Emine Y (2002). The influence of functional forces on the biomechanics of implant-supported prostheses—a review. J Dent.

[18] Balshi T, Hernandez R, Pryszlak M, Rangert B (1996). A comparative study of one implant versus two replacing a single molar. Int J Oral Maxillofac Implants.

[19] Sullivan D, Siddiqui A (1994). Wide diameter implants: overcoming problems. Dent Today.

[20] Bahat O, Handelsman M (1996). Use of wide implants and double implants in the posterior jaw: a clinical report. Int J Oral Maxillofac Implants.

[21] Petropoulos V, Wolfinger G, Balshi T (2004). Complications of mandibular molar replacement with a single implant: a case report. J Can Dent Assoc.

[22] Jackson BJ (2011). Small diameter implants: specific indications and considerations for the posterior mandible: a case report. J Oral Implantol.

[23] Misch C (2008). Contemporary implant dentistry.

[24] Von Recum A (1986). Handbook of biomaterials evaluation: scientific, technical and clinical testing of implant materials.

[25] Shigley J, Mischke C (1989). Mechanical engineering design.

[26] Bidez M, Misch C (1992). Issues in bone mechanics related to oral implants. Implant Dent.

[27] Sevimay M, Turhan F, Kiliçarslan M, Eskitascioglu G (2005). Three-dimensional finite element analysis of the effect of different bone quality on stress distribution in an implant-supported crown. J Prosthet Dent.

[28] Bidez M, Misch C (2005). Clinical biomechanics in implant dentistry.

[29] Misch C, Suzuki I, Misch-Dietch D (2005). A positive correlation between occlusion between occlusal trauma and peri-implant bone loss -literature support. implant dent.

[30] Misch C (1999). Implant design considerations for the posterior regions of the mouth. Implant Dent.

[31] Himmlova L, Dostalova T, Kacovsky A, Konvickova S (2004). Influence of implant length and diameter on stress distribution: a finite element analysis. J Prosthet Dent.

[32] Shetty S, Puthukkat N, Bhat S, Shenoy K (2014). Short implants: a new dimension in rehabilitation of atrophic maxilla and mandible. Journal of Interdisciplinary Dentistry.

[33] Misch C, Bidez M (1999). Contemporary implant dentistry.

[34] Misch C (2008). Implant body size: a biomechanical and esthetic rationale.

[35] O'Mahony A, Bowles Q, Woolsey G, Robinson S, Spencer P (2000). Stress distribution in the single-unit osseointegrated dental implant: finite element analyses of axial and off-axial loading. Implant Dent.

[36] Fawzi S (2013). The effect of dental implant design on bone induced stress distribution and implant displacement. Int. J. Comput. Appl..

[37] Flanagan D (2011). Avoiding osseous grafting in the atrophic posterior mandible for implant-supported fixed partial dentures: a report of 2 cases. J Oral Implantol.

[38] Seong W, Korioth T, Hodges J (2000). Experimentally induced abutment strains in three types of single-molar implant restorations. J Prosthet Dent.

[39] Jarvis W (1997). Biomechanical advantage of wide-diameter implants. Compend Contin Educ Dent.

[40] Rangert B, Jemt T, Jörnéus L (1989). Forces and moments on Brånemark implants. Int J Oral Maxillofac Implants.

[41] Misch CE (2005). A scientific rationale for dental implant design.

[42] Misch C (1993). Occlusal considerations for implant-supported prostheses.

[43] Rangert B (1996). Biomechanical considerations when choosing a platform. Nobel Biocare Global Forum.

[44] Linish V, Peteris A (2003). Restorative factors that affect the biomechanics of the dental implant. Stomatologija, Baltic Dental and Maxillofacial Journal.

[45] Skalak R. Aspects of biomechanical considerations. In: Branemark PI, Zarb GA, Albrektsson, eds. Tissue integrated prostheses. 1985: p. 117–28.

[46] Davis D, Rimrott R, Zarb G (1988). Studies on frameworks for osseointegrated prostheses: part 2. The effect of adding acrylic resin or porcelain to form the occlusal superstructure. Int J Oral Maxillofac Implants.

[47] Gracis S, Nicholls J, Chalupnik J, Yuodelis R (1990). Shock-absorbing behavior of five restorative materials used on implants. Int J Prosthodont.

[48] Skalak R (1983). Biomechanical considerations in osseointegrated prostheses. J Prosthet Dent.

[49] Misch C (2008). Clinical biomechanics in implant dentistry.

[50] Lundgren D, Laurell L (1994). Biomechanical aspects of fixed bridgework supported by natural teeth and endosseous implants. Periodontol 2000.

[51] Duyck J, Van Oosterwyck H, Vander SJ, De Cooman M, Puers R, Naert I (2000). Influence of prosthesis material on the loading of implants that support a fixed partial prosthesis: in vivo study. Clin Implant Dent Relat Res.

[52] Stegaroiu R, Kusakari H, Nishiyama S, Miyakawa O (1998). Influence of prosthesis material on stress distribution in bone and implant: a 3-dimensional finite element analysis. Int J Oral Maxillofac Implants.

[53] Stegaroiu R, Khraisat A, Nomura S, Miyakawa O (2004). Influence of superstructure materials on strain around an implant under 2 loading conditions: a technical investigation. Int J Oral Maxillofac Implants.

[54] Desai S, Singh R, Karthikeyan I, Reetika J (2012). Three-dimensional finite element analysis of effect of prosthetic materials and short implant biomechanics on D4 bone under immediate loading. J Dent Implant.

[55] Sertgoz A (1997). Finite element analysis study of the effect of superstructure material on stress distribution in an implant-supported fixed prosthesis. Int J Prosthodont.

[56] Wang T, Leu L, Wang J, Lin L (2002). Effects of prosthesis materials and prosthesis splinting on peri-implant bone stress around implants in poor-quality bone: a numeric analysis. Int J Oral Maxillofac Implants.

